# Accuracy of Edentulous Computer-Aided Implant Surgery as Compared to Virtual Planning: A Retrospective Multicenter Study

**DOI:** 10.3390/jcm9030774

**Published:** 2020-03-12

**Authors:** R. Vinci, M. Manacorda, R. Abundo, A.G. Lucchina, A. Scarano, C. Crocetta, L. Lo Muzio, E.F. Gherlone, F. Mastrangelo

**Affiliations:** 1 Dental Clinic, Ateneo Vita Salute, San Raffale Milan, 20019, Italy; vinci.raffaele@unisr.it; 2 Private Practice, 20021 Milan, Italy; manacorda.michele@unisr.it; 3 Private Practice, 10121 Turin, Italy; robabund@yahoo.it; 4 Department of Oral and maxillofacial Sciences, University of Piemonte Orientale, 10121 Turin, Italy; arlucchina@gmail.com; 5 Department of Medical, Oral and Biotechnological Sciences, University of Chieti, 66100 Chieti, Italy; ascarano@unich.it; 6 Department of Economics, University of Foggia, 71121 Foggia, Italy; corrado.crocetta@unifg.it; 7 Department of Clinical and Experimental Medicine, University of Foggia, 71122 Foggia, Italy; lorenzo.lomuzio@unifg.it; 8 Dental Clinic, Ateneo Vita Salute, San Raffale Milan, Italy; gherlone.enrico@unisr.it

**Keywords:** guided surgery, titanium dental implant, Three-Dimensional implant accuracy, Three-Dimensional software implant, planning, permutation tests, non-parametric combination, multivariate analysis, osseointegration, immediate-load dental implants

## Abstract

Purpose: To evaluate the accuracy of computer-aided dental implant positions obtained with mucosal-supported templates as compared to Three-Dimensional (3D) planning. Materials and methods: One-hundred implants were inserted into 14 edentulous patients using the All-on-4/6 protocol after surgical virtual planning with RealGUIDE, 3DIEMME, and Geomagic software. After 6 months, three-dimensional neck (V) and apex (S) spatial coordinates of implants and angle inclination displacements as compared to virtual plans were evaluated. Results: The S maxilla coordinates revealed a significant discrepancy between clinical and virtual implant positions (*p*-value = 0.091). The V coordinates showed no significant differences (*p*-value = 0.71). The S (*p*-value = 0.017) and V (*p*-value = 0.038) mandible coordinates showed significant discrepancies between the clinical and virtual positions of the screws. Implant evaluation showed a 1-mm in average of the horizontal deviation in the V point and a 1.6-mm deviation in the S point. A mean 5° angular global deviation was detected. The multivariate permutation test of the S (*p*-value = 0.02) confirmed the difference. Greater errors in the mandible were detected as compared to the maxilla, and a higher S discrepancy was found in the posterior jaw compared to the anterior section of both the mandible and maxilla. Conclusions: Computer-aided surgery with mucosal-supported templates is a predictable procedure for implant placement. Data showed a discrepancy between the actual dental implant position as compared to the virtual plan, but this was not statistically significant. However, the horizontal and angle deviations detected indicated that flap surgery should be used to prevent implant positioning errors due to poor sensitivity and accuracy in cases of severe jaw atrophy.

## 1. Introduction

Immediate implant-supported prosthetic rehabilitation is considered to be a routine procedure with a high success rate [1,2,3]. Presently, Three-Dimensional (3D) preoperative planning through cone beam computed tomography analysis (CBCT) allows for the verification of the maxillary anatomy and qualitative and quantitative bone structures before dental implant placement [4,5]. The CBCT Standard Triangulation Language (STL) files elaborated through specific surgical software, together with stereolithographic (SL) models, promote individual virtual planning [6] that allows an immediate prosthetic loading of the implants with a reduction in surgical times, increasing the comfort of the patients [7,8] and the predictability of surgical results [9,10]. In the literature, computer-aided surgical systems have shown high accuracy rates in terms of implant position, depth, and angle, avoiding intraoperative surgical complications and poor positioning of implants which can compromise the primary stability and/or success of immediate-load restoration techniques [11,12,13]. However, in a recent systematic review of static navigation systems, Tahmeseb A. et al. found a 1.2-mm (1.04–1.44 mm) mean horizontal deviation at the coronal entry point and a 1.48-mm (1.28–1.58 mm) deviation in the apical endpoint, respectively, with a mean angular deviation of 3.5° (3.0–3.96°) [14]. However, numerous variables have been found to affect virtual planning and the in vivo position of the dental implant [15]. Thus, it is necessary to evaluate the accuracy of the virtual and clinical measurements of lengths and angles [16] due to the many errors related to the different phases of data acquisition, as well as to data elaboration during digital and surgical workflow [17]. Many studies have been carried out to compare the planned position of dental implants with their actual insertion position in the maxilla, using implant neck/apex points or 3D coordinates (X, Y, Z axes) to analyze the sensitivity of the software and to determine predictability of the results. The aim of the study was to evaluate the accuracy of the virtual computer-aided approach in the overall digital workflow using mucosal-supported templates Computer-Aided Design and Manufacturing (CAD-CAM) as compared to the clinical position of immediate-load dental implants in atrophic areas of bone in totally edentulous patients.

## 2. Materials and Methods

### 2.1. Patient Selection

Fourteen patients (6 male/8 female; mean age 58) were selected from four different private dental clinics from November 2014 to July 2017. All presented with good health, total edentulism of the jaws, and the need for dental implant rehabilitation. Inclusion criteria were: an upper opening of the mouth wider than 50 mm, edentulism of the maxilla arches, and sufficient bone available for positioning the implant fixture. The exclusion criteria were: cardiac disease, chemotherapy, radiotherapy, bisphosphonate therapy, pregnancy, asthmatic problems, decompensated diabetes, smoking habit (more than 10 cigarettes/day), and maxillary parafunctions. No bone regeneration was requested. In order to reduce the study variables, all surgeons had the same level of expertise and used the same digital work flow for the computer-guided surgery, with the same 3D device and the same implant system. All patients were examined through preliminary CBCT analysis (NewTom Evo - NewTom, Verona, Italy) using a prosthesis with a radiopaque marker, and underwent standardized CBCT scanning with an interocclusal index. To complete the digital data, some optical scans (Activity 885, Smart Optics, Bochum, Germany) were performed. The 3D STL files were imported into 3Diagnosys (3DIEMME, Italy) to match the CBCT Dicom data and to perform virtual planning (RealGUIDE, 3DIEMME, Milan, Italy). All data were collected using software with an adapting algorithm, called “best-fit”, to optimize the anatomic curves of the maxillo-facial complex as well as the regular geometry of the radiological markers. After digital image segmentation, the virtual implants were placed in the optimal position according to the anatomy and prosthetic planning. The STL data were processed with the Plasty-CAD-3DIEMME system (DWS 20D, DWS SYSTEMS, Bergamo Italy) and surgical guide templates were created with mucous support and a stabilizing system with bi-cortical bone fixation pins. After administration of local anesthetic (4% articaine with 1: 200.000 adrenaline), the surgical guide was positioned on the maxillary arch of the patients and anchored by three bi-cortical bone pins. After flapless surgery, only Winsix TTx implants (diameter: 3.8 or 4.5 mm) with external hexagonal connections were placed using the “Just on-four” or “Just on-six” techniques. All implants were inserted with 35–55 N/m torque and immediately loaded with a provisional prosthesis. (Table 1)

### 2.2. Computerized and Statistical Analysis

**Figure 1:**
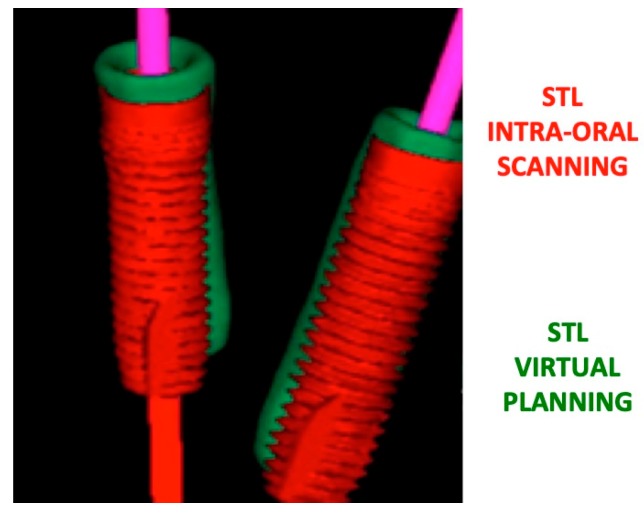
The 3D spatial software reconstruction of dental implant screws in pre-surgical virtual planning vs actual clinical position after the surgical procedure.

The control CBCT procedure was used to verify the differences between the clinical implant fixture position after the surgery as compared to that in virtual planning. Geomagic Studio software (3D SYSTEM, Geomagic, Morrisville, North Carolina -USA) was used. For the evaluation of the three spatial coordinates (x_v_, y_v_, z_v_ e x_s_, y_s_, z_s_) of each dental implant, the angles formed by the axes of inclination between the implants were calculated. (Table 2) The neck (V) and the apex (S) points were chosen to understand the accuracy and the sensitivity of virtual planning as compared with the clinical results. To evaluate the three-dimensional spatial position (x, y, z) of single implants (Figure 1) post-surgery, the CBCT analysis used a specific algorithm to minimize the relative distance according to an interactive process of the files matching, guaranteeing precise and repeatable overlaying. Following the alignment process, a mean error value was calculated using the distance of the points between the two surfaces (from the STL and the DICOM files), which were considered acceptable when less than 0.1–0.15 mm (on the entire arch). To calculate the Euclidean distances (S and V), the formulas $=\sqrt{\left(x_{s}^{2}+y_{s}^{2}+z_{s}^{2}\right)}$ and $V=\sqrt{\left(x_{v}^{2}+y_{v}^{2}+z_{v}^{2}\right)}$ were used. The Smean and Vmean were calculated for each implant of each patient, wher were used. Therefore, Smean and Vmean were calculated for each implant of each patient where

(1) $$S_{mean}=\frac{\sum_{i=1}^{n}S_{i}}{n},\;V_{mean}=\frac{\sum_{i=1}^{n}V_{i}}{n}$$

**Figure 2:**
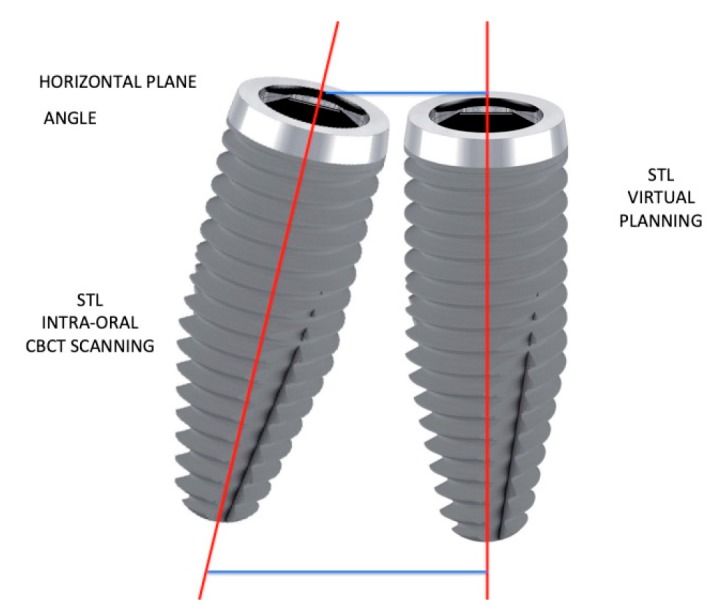
The 3D STL intra-oral cone-beam analysis (CBCT) scanning position data as compared to the STL virtual planning data in millimetric horizontal and spatial angle evaluation. Software reconstruction of dental implant virtual planning vs clinical position after the surgical procedure.

**Figure 3:**
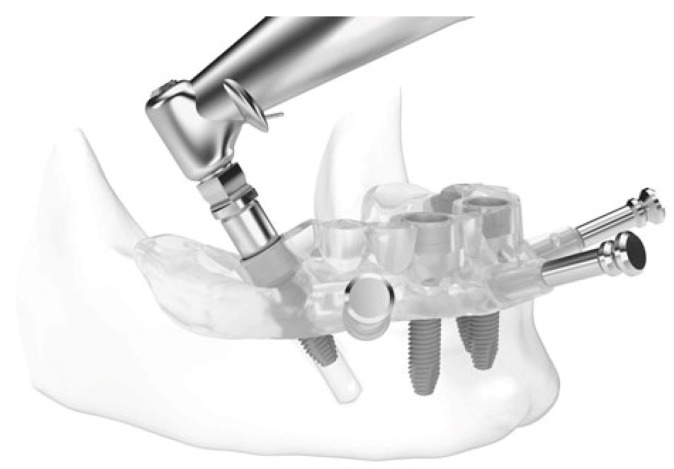
Just-on-4 surgical work-flow with a mucosa-supported template.

All measurements were repeated three times, by two blinded researchers to evaluate the reliability and reproducibility of the records. A non-parametric permutation method was used to data collected to establish a null hypothesis for all entitles of disturbance, including the P distribution. These tests do not depend on the type of distribution of the population, they are not based on the parameters of the distribution and it is possible to apply them even in cases of qualitative data. This tests are suggested when the variables do not have a Gaussian distribution (they are very asymmetric or present more than one peak); the sample is small; the observations are of ordinal type. The mean differences were evaluated by the permutation test, comparing the planned position and the real one. To obtain a multivariate and global result for both the Euclidean distances, as every implant was interested by different variables, the non-parametric combination (NPC) of partially dependent tests was adopted [15,18]. Two distinct permutation tests, for S and for V, that were combined with an adequate combination function. Tests were carried out separately but simultaneously, resulting in a global test. To be able to jointly consider the two variables, considering the structure of dependence between them (unknown), it is necessary to base the two tests of permutation on the same entire permutation. Figure 2 and Figure 3 explained for the no surgeons, and the allon 4 implants surgical procedure in the mandible sites.

## 3. Results

100 dental implants immediate loaded were placed in 14 total edentulous maxilla with using a surgical template mucosa supported. In 12 upper jaws a total of 51 implants were placed, 23 implants in the anterior area and 28 in the posterior area. While in 11 mandibles total 49 implants were placed, 23 in the anterior and 26 in the posterior area. The sites 15, 25, and 42 were most treated with 11, 10, and 9 implants respectively. (Table 1) No intraoperative complications and no implant failure after 1 yr follow-up were detected. All virtual planning data were matched with the CBCT-STL post-surgery porcedure and the implant fixture showed an error value. In maxillary area, 0.30 mm (range: 0.10–1.57 mean 0.89) S discrepancy and 0.37 mm (range: 0.30–1.77 mean 0.67) V discrepancy between virtual planning to clinical result were detected. In mandible implant sites, S difference 0,43 mm (range: 0.30–1.77 mean 0.31) and V difference 0.28 mm (range: 0.08–1.18 mean 0.12) were observed. The implant S discrepancy global mean value was 0.43 mm (range: 0.10–2.02 mean 0.75) and V mean value showed 0.35 mm (range: 0.08–1.77 mean 0.56). In the anterior area the S discrepancy between virtual planning to clinical result was 0.44 mm (range: 0.17–2.66 mean 0.88) while V value was 0.31 mm (range: 0.08–1.30 mean 0.41). In the posterior area 0.40 mm (range: 0.10–3.54 mean 0.79) S discrepancy and 0.38 mm (range: 0.27–1.77 mean 0.31) at V point were detected (Table 2). Global *p*-value = 0.001 of the multivariate permutation test was detect to study differences between the two Euclidean distances (virtual and real). 

The partial coordinates were examined $x_{s},\;y_{s},$ and $z_{s}$ and $x_{v},\;y_{v},$ and $z_{v}$, separately for S and V. Multivariate S coordinates permutation test detect global *p* value = 0.02 confirming the difference between the virtual and real implant positions. Significant difference was related to the x_s_ coordinate: *p*(x) = 0.001; *p*(y) = 0.43; *p*(z) = 0.98. Non-significant global *p*-value (= 0.12) resulted in V coordinates while a significant difference on the coordinates x_v_: *p*(x) = 0.036; *p*(y) = 0.84; *p*(z) = 0.2 is found. In both arches the permutation test confirmed the results obtained with the entire sample of implants (global *p* value = *p*_s_ = *p*_v_ = 0.001). In the maxilla, for S coordinates a significant level was obtained with a global *p* value = 0.091 (x_s_: *p*(x) = 0.068; *p*(y) = 0.28; *p*(z) = 0.20). For the V coordinates no significant difference was found with global *p* value = 0.71 (*p*(x) = 0.22; *p*(y) = 0.85; *p*(z) = 0.89). In the mandible, for the S coordinate a significant global p value = 0.017 was obtained (x_s_: *p*(x) = 0.002; *p*(y) = 0.95; *p*(z) = 0.17). For the V coordinates, the global p value was significant (0.038) above all for the coordinate z_v_: *p*(x) = 0.063; *p*(y) = 0.92; *p*(z) = 0.021. The entire sequence of the implants was divided into two groups according to the position the anterior (positions 1,2,3) or in the posterior (positions 4,5,6) of the mouth. After applying the multivariate permutation test on the S and V variables, the results obtained were confirmed for the sample of all the implants global *p* value = *p*(S) = *p*(V) = 0.001) in anterior sector as well as the posterior one. Examining the coordinates of the apex a nonsignificant global difference was found (global *p* value = 0.23), but on examination of the partial coordinates a significant difference was found for the coordinates x_s_: *p*(x) = 0.02; *p*(y) = 0.87; *p*(z) = 0.96. No evidence was obtained in V coordinates (global *p* value = 0.13). In the posterior area, the S coordinate showed a 10% global difference (global *p* value = 0.065). In partial coordinates a significant difference was found for the following coordinates x_s_: *p*(x) = 0.007; *p*(y) = 0.35; *p*(z) = 0.99. For the implant V coordinates no significant difference was found for the global test as well as for the partial ones: global *p* value = 0.35; *p*(x) = 0.44; *p*(y) = 0.35; *p*(z) = 0. 31.(Table 3 and Table 4).

## 4. Discussion

Computer-aided systems are valid tools for flapless virtual planning surgery using presurgical CBCT data implementation, with specific software to reduce postoperative morbidity and to improve patient compliance. The absence of surgical flap incisions, the pre-determination of the implant positions and fixture parameters, the control of cutter depth and stitching, and the previsualization and production of protheses before surgery for immediate loading of implants contribute to a reduction in surgical time and errors. Moreover, the accuracy of guided surgery systems must be carefully studied in order to respond to the growing needs of patients and increase the predictability of surgical results and the success of treatments. In computer-aided implant surgery, the absence of incisions and flaps and the pre-determination of exact implant positions and the depths and the sequences of the drills contribute to reducing the surgical time and patient discomfort. However, this technique has some disadvantages, including potential damage to the bone due to insufficient irrigation and the inability to visualize the surgical anatomical landmarks, with increased risk of error in implant positioning with increasing degrees of maxillary bone atrophy. Many studies have been conducted to assess the accuracy of virtual implant planning, and in all cases a discrepancy has been demonstrated between the virtual plan and the actual position of the implant in the oral cavity at the end of the surgery. The surgical problems with implant fixture are relative to the deviations in the three spatial dimensions of the screw position, which are higher in horizontal points and lower in the vertical points, as described in the literature, confirming that implant rehabilitation through computer-aided surgery is a predictable procedure that requires constant verification, especially in flapless surgery, to reduce the high risk of error in implant positioning during surgical procedures that use supported surgical templates. The first crucial factor affecting clinical result accuracy is the stability of the surgical template during the CBCT analysis and during surgical procedures with respect to surgical template positioning on the bone with pins to avoid damaging noble anatomic structures such as nerves, vessels, etc., because any small deviations may cause surgical errors and iatrogenic anatomical lesions, which are reported in the literature to occur in 9.1% of all cases. The second crucial factor affecting computer-aided surgery accuracy for the correct angle of insertion of the implant drills, is relates to the area of surgery and the mouth-opening capacity because in 2.3% of cases in the posterior maxillary area there is a limited interocclusal distance [15]. The third crucial factor that affects accuracy is related to the bone volume and bone architecture in atrophic bone areas of the jaws, together with potential micromovements of the surgical mucosa-supported template due to the typical resilience of the oral mucosa [15,16]. Mucosa-supported guided surgery is a tool used to preoperatively study the anatomical conditions of the jaws, pre-view the best implant insertion method, and plan the implant dimensions integrated into the predictable prosthetic rehabilitation workflow to prepare immediate-load provisional restoration, avoiding complications [17,18]. At present, mucosa-supported guided surgery scientific literature is scarce and more in vitro and vivo studies should be carried out to understand surgical accuracy. [19] A recent review and meta-analysis compared three types of guides (bone, mucosa, or tooth-supported), with a total 345 implants placed with mucosa-supported guides, concluding that more clinical studies should be performed to provide evidence about the accuracy of guided surgery, as well as to evaluate the variables that could affect the precision of the technique. [20] In the literature, few scientific studies have correlated implant dimensions and bone density with guided surgery implant placement accuracy [21], and there are only simple theoretical references to bone density, mouth opening, visibility, surgical guide stability, skill of the surgeon, and patient movements during surgical procedures. [22] However, during bone drilling and implant insertion, mucosa-supported guided surgery is a blind technique, and thus to obtain predictable clinical results, presurgical 3D implant planning should be highly accurate in order to translate the virtual planning data into clinical surgical practice and to place the implants in the correct prosthetic positions, avoiding damage to important anatomic structures such as nerves, roots, or maxillary sinuses. [23] In 2014, Schneider et al. showed that metal sleeves with a degree of tolerance to the surgical templates allowed cutter entrance and movement during surgical procedures. This specific tolerance could make them deviate during the drilling procedures, altering the angle of surgical site formation and final implant placement into the bone. Furthermore, long cutters promote greater angular or horizontal deviations. Thus, the length of the implants could also influence the accuracy of the clinical result [24]. Recently, Hoffmann et al. [25] reported statistically significant differences in implant surgical computer-aided accuracy, with mean angular deviations of 4.2 ± 1.8 and 11.2 ± 5, respectively. In 2018, Chang-Kai et al. reported 1.50 ± 0.79 mm horizontal deviation values at the apical endpoint. Implant placement accuracy with computer-aided static navigation systems was shown to be better (6.02 ± 3.71) compared to the manual implant placement (9.26 ± 3.62) [12,26]. Augmented reality devices could be used to display the virtual planning image as compared to the reality of the surgical field [12]. In a recent study, Vercruyssen et al. [27] showed a high accuracy error of 0.9 mm (range: 0.1–4.5, mean 0.8) at the neck and of 1.2 mm (range: 0.2–4.9, mean 1.1) at the apex, with an angular deviation error of 2.7 (range: 0.0–6.6, mean 2.3). In 2016, Schneider described accuracy through an evaluation of implant apex deviation in terms of height, showing a 1.07 mm deviation at the neck point, a 1.63-mm deviation at the apex, and a 5.26 angular discrepancy [15]. Our study confirmed a risk of error between the virtual plan and the clinical implant position in surgical mucosa-supported templates, however with lower values than those found in the scientific literature. Indeed, the data obtained showed a 0.30 m discrepancy at the apex with a 0.37 mm global horizontal discrepancy at the neck in the maxilla, and a 0,43-mm discrepancy at the apex with a 0.28 mm global horizontal discrepancy at the neck in the mandible. A 0.43 mm global mean value at the apex and a 0.35 mm discrepancy at the neck were detected. The multivariate scale permutation test confirmed the accuracy discrepancy in 3D spatial coordinates. Significant differences were shown in the mandible at the apex (*p* = 0,017), while higher values were found in the posterior area than the anterior for the apex (*p* = 0,001) and for the angle in the maxillary area. In conclusion, computer-aided surgery with a mucosa-supported template seems to be a predictable and reproducible procedure capable of reducing surgical times and patient discomfort [28,29,30,31]. However, our study has shown an inaccuracy of the virtual projection, with a horizontal and angle discrepancy between the clinical position of the dental implant as compared to virtual planning. Although virtual planning reproduces in detail the anatomical and clinical characteristics of the future implant site, it should be noted that the accuracy required within the flapless surgical procedure does not seem to be sufficient in cases of severe atrophy of the jaws and in the presence of particular anatomies of the maxillary bone and in the posterior areas of the maxilla, with risks of error present in the procedure.

## Figures and Tables

**Table 1:** The 100 dental implants in the maxilla or mandible site positions.

Arch	Site	Implants Numbers
Anterior Maxilla	11	4
	12	6
	13	2
	21	2
	22	6
	23	3
Posterior Maxilla	14	3
	15	11
	24	3
	25	10
	26	1
Anterior Mandible	31	3
	32	8
	33	1
	42	9
	43	2
Posterior Mandible	34	4
	35	6
	36	3
	44	5
	45	6
	46	2
		Total 100

**Table 2:** The horizontal apex (S) and neck (V) implant discrepancies.

Implant Discrepancy	APEX (S) mm	NECK (V) mm
Maxillary sites	0.30 mm (range: 0.10–1.57 mean 0.89	0.37 mm (range 0.30–1.77 mean 0.67)
Mandible sites	0.43 mm (range: 0.30–1.77 mean 0.31	0.28 mm (range: 0.08–1.18 mean 0.12
Anterior area	0.44 mm (range: 0.17–2.66 mean 0.88	0.31 mm (range: range: 0.08–1.30 mean 0.41)
Posterior area	0.40 mm (range: 0.10–3.54 mean 0.79	0.38 mm (range: 0.27–1.77 mean 0.31
Global mean value	0.43 mm (range: 0.10–2.02 mean 0.75	0.35 mm (range: 0.27–1.77 mean 0.56

**Table 3:** Statistical 3D evaluation of discrepancies between the clinical and virtual implant positions in single patients using multivariate permutation tests.

Patients	S mean Value	Y mean Value	Global Mean
1	0.579	0.461	2.51
2	0.800	0.506	2.49
3	0.732	0.685	2.30
4	0.967	0.732	3.73
5	0.952	0.699	2.63
6	0.872	0.665	2.44
7	1.043	0.990	1.91
8	1.175	0.856	2.64
9	0.845	0.796	2.53
10	1.060	0.551	2.00
11	0.834	0.624	2.44
12	0.802	0.778	2.86
13	0.920	0.770	4.74
14	0.767	0.692	1.11

**Table 4:** Statistical 3D evaluation of discrepancies using multivariate permutation tests in maxillae vs mandibles and anterior vs posterior areas using clinical and virtual apex (S) and neck (V) implant positions.

Distance/Coordinates	Apex (S)	Neck (V)
MAXILLA		
Euclidean Distance	0.09	0.71
x	0.07	0.22
y	0.28	0.85
z	0.20	0.89
MANDIBLE		
Euclidean Distance	0.02	0.04
x	0.00	0.06
y	0.95	0.92
z	0.17	0.02
ANTERIOR AREA		
Euclidean Distance	0.23	0.13
x	0.02	0.03
y	0.87	0.46
z	0.96	0.54
POSTERIOR AREA		
Euclidean Distance	0.07	0.35
x	0.01	0.44
y	0.35	0.35
z	0.99	0.31
FULL MOUTH		
Euclidean Distance	0.02	0.12
x	0.00	0.04
x	0.43	0.84
z	0.98	0.20

## References

[1] Hämmerle C.H.F., Jung R.E., Feloutzis A. (2002). A systematic review of the survival of implants in bone sites augmented with barrier membranes (guided bone regeneration) in partially edentulous patients. J. Clin. Periodontol..

[2] Pjetursson B.E., Bragger U., Lang N.P., Zwahlen M. (2007). Comparison of survival and complication rates of tooth-supported fixed dental prostheses (fdps) and implant-supported fdps and single necks (scs). Clin. Oral Implant. Res..

[3] Jung R.E., Pjetursson B.E., Glauser R., Zembic A., Zwahlen M., Lang N.P. (2008). A systematic review of the 5- year survival and complication rates of implant-supported single neck. Clin. Oral Implat. Res..

[4] Schulze D., Heiland M., Thurmann H., Adam G. (2004). Radiation exposure during midfacial imaging using 4- and 16-slice computed tomography, cone beam computed tomography systems and conventional radiography. Dentomaxillofacial Radiol..

[5] Guerrero M.E., Jacobs R., Loubele M., Schutyser F., Suetens P., Van Steenberghe D. (2006). State-of-the-art on cone beam CT imaging for preoperative planning of implant placement. Clin. Oral Investig..

[6] Jung R.E., Schneider D., Ganeles J., Wismeijer D., Zwahlen M., Hämmerle C.H.F., Tahmaseb A. (2009). Computer technology applications in surgical implant dentistry: A systematic review. Int. J. Oral Maxillofac. Implant..

[7] Chen X., Yuan J., Wang C., Huang Y., Kang L. (2009). Modular Preoperative Planning Software for Computer-Aided Oral Implantology and the Application of a Novel Stereolithographic Template: A Pilot Study. Clin. Implant. Dent. Relat. Res..

[8] Tahmaseb A., Wismeijer D., Coucke W., Derksen W. (2014). Computer technology applications in surgical implant dentistry: A systematic review. Int. J. Oral Maxillofac. Implant..

[9] Kupeyan H.K., Shaffner M., Armstrong J. (2006). Definitive CAD/CAM-guided prosthesis for immediateloading of bone-grafted maxilla: A case report. Clin. Implan. Dent. Relat. Res..

[10] Hämmerle C., Stone P., Jung R.E., Kapos T., Brodala N. (2009). Consensus statements and recommended clinical procedures regarding computer-assisted implant dentistry. Int. J. Oral Maxillofac. Implant..

[11] Gargallo-Albiol J., Barootchi S., Salomó-Coll O., Wang H.-L. (2019). Advantages and disadvantages of implant navigation surgery. A systematic review. Ann. Anat. - Anat. Anz..

[12] Alfonso Mediavilla G., Elena Riad D., Álvaro Z.-M., Rubén A.-P., Sofía Hernández M. (2019). Accuracy of Computer-Aided Dynamic Navigation Compared to Computer-Aided Static Navigation for Dental Implant Placement: An in Vitro Study. J. Clin. Med..

[13] Widmann G., Bale R. (2006). Accuracy in computer-aided implant surgery--a review. Int. J. Oral Maxillofac. Implant..

[14] Tahmaseb A., Wu V., Wismeijer D., Coucke W., Evans C., Wu V. (2018). The accuracy of static computer-aided implant surgery: A systematic review and meta-analysis. Clin. Oral Implant. Res..

[15] Schneider D., Marquardt P., Zwahlen M., Jung R.E. (2009). A systematic review on the accuracy and the clinical outcome of computer-guided template-based implant dentistry. Clin. Oral Implant. Res..

[16] D’Haese J., Van De Velde T., Elaut L., De Bruyn H. (2009). A Prospective Study on the Accuracy of Mucosally Supported Stereolithographic Surgical Guides in Fully Edentulous Maxillae. Clin. Implant. Dent. Relat. Res..

[17] Ruth V., Kolditz D., Steiding C., Kalender W.A. (2017). Metal Artifact Reduction in X-ray Computed Tomography Using Computer-Aided Design Data of Implants as Prior Information. Investig. Radiol..

[18] Tahmaseb A., De Clerck R., Eckert S., Wismeijer D. (2011). Reference-based digital concept to restore partially edentulous patients following an immediate loading protocol: A pilot study. Int. J. Oral Maxillofac. Implant..

[19] Vercruyssen M., Laleman I., Jacobs R., Quirynen M. (2015). Computer supported implant planning and guided surgery: A narrative re- view. Clin. Oral Implant. Res..

[20] Gallardo Y.N.R., Silva-Olivio I.R.T., Mukai E., Morimoto S., Sesma N., Cordaro L. (2016). Accuracy comparison of guided surgery for dental implants according to the tissue of support: A systematic review and meta-analysis. Clin. Oral Implant. Res..

[21] Cassetta M., Di Mambro A., Giansanti M., Stefanelli L.V., Cavallini C. (2013). The intrinsic error of a stereolithographic surgical template in im- plant guided surgery. Int. J. Oral Maxillofac. Surg..

[22] Van Assche N., Quirynen M. (2010). Tolerance within a surgical guide. Clin. Oral Implant. Res..

[23] Andoni J. (2018). Accuracy of mucosa supported guided dental implant surgery. Clin. Case Rep..

[24] Schneider D., Schober F., Grohmann P., Hämmerle C.H.F., Jung R.E. (2014). In-vitro evaluation of the tolerance of surgical instruments in templates for computer-assisted guided implantology produced by 3-D printing. Clin. Oral Implant. Res..

[25] Hoffmann J., Westendorff C., Reinert S., Gomez-Roman G. (2005). Accuracy of navigation-guided socket drilling before implant installation compared to the conventional free-hand method in a synthetic edentulous lower jaw model. Clin. Oral Implant. Res..

[26] Chen C.-K., Yuh D.-Y., Huang R.-Y., Fu E., Tsai C.-F., Chiang C.-Y. (2018). Accuracy of Implant Placement with a Navigation System, a Laboratory Guide, and Freehand Drilling. Int. J. Oral Maxillofac. Implant..

[27] Vercruyssen M., Cox C., Naert I., Jacobs R., Teughels W., Quirynen M. (2015). Accuracy and patient-centered outcome variables in guided implant surgery: A RCT comparing immediate with delayed loading. Clin. Oral Implant. Res..

[28] Pesarin F., Salmaso L. (2010). Permutation Tests for Complex Data.

[29] Antonucci L., Bolzan M., Carrozzo E., Crocetta C., Di Gioia L., Manacorda M., Mastrangelo F., Russo M., Salmaso L. (2019). Accuracy of computer guided implant dentistry: A permutation testing approach. Electron. J. Appl. Stat. Anal. (EJASA).

[30] Bover-Ramos F., Cervera-Ballester J., Viña-Almunia J., Peñarrocha-Diago M., García-Mira B. (2018). Accuracy of Implant Placement with Computer-Guided Surgery: A Systematic Review and Meta-Analysis Comparing Cadaver, Clinical, and In Vitro Studies. Int. J. Oral Maxillofac. Implant..

[31] Wenjuan Z., Zhounghao L., Lianshenng S., Chia-Ling K., David M.S. (2018). Clinical Factors affecting the accuracy of guided implant surgert- A systematic review and meta-analysis. J. Evid. Based Dent. Pr..

